# A short review of drug–food interactions of medicines treating overactive bladder syndrome

**DOI:** 10.1007/s11096-016-0383-5

**Published:** 2016-10-13

**Authors:** Paweł Paśko, Tomasz Rodacki, Renata Domagała-Rodacka, Danuta Owczarek

**Affiliations:** 1 Department of Food Chemistry and Nutrition, Faculty of Pharmacy, Jagiellonian University Medical College, Kraków, Medyczna 9, 30-688 Kraków, Poland; 2 Department of Gastroenterology, Hepatology and Infectious Diseases, Faculty of Medicine, Jagiellonian University Medical College, 31-531 Kraków, Poland

**Keywords:** Anticholinergic drugs, Drugs–food interactions, Overactive bladder syndrome, Review, Urinary incontinence

## Abstract

*Background* Overactive bladder syndrome is a condition where one or more of the symptoms such as pollakiuria, urgent need to urinate, nocturia and urinary incontinence is observed. Its prevalence ranges between 7 and 27 % in men and 9–43 % in women. The role of a pharmacist is to educate the patient on medications administration scheme, and drug interactions with particular food or food components. *Aim of the review* To assess a potential impact of food and fruit juice on the pharmacokinetic and therapeutic effects of medications used in treating overactive bladder syndrome. This information will enhance pharmaceutical care and is vital and helpful for pharmacists counseling their patients. *Method* In order to gather information on interactions of medications employed in bladder dysfunctions, the English language reports published in the PubMed, Embase, Cochrane and CINAHL database over the years 1996–2015 were studied. Additionally, other resources, namely drugs.com, Medscape, UpToDate, Micromedex, Medical Letter, as well as Stockley Drugs Interaction electronic publication were included in the study. The analysis also covered product data sheets for particular medicinal products. *Results* Meals and the consumption of grapefruit juice were found to exert a diversified effect on the pharmacokinetics of drugs employed in overactive bladder syndrome therapy. Neither tolterodine, nor mirabegron interact with food and citrus fruit juice, whereas darifenacin, fesoterodine, oxybutynin and solifenacin do interact with grapefruit and others citrus fruit juice. The effects of such interactions may potentially be negative to patients. Trospium absorption is significantly decreased by food. *Conclusion* For selected medicines used in treating bladder dysfunctions food and grapefruit juice consumption may significantly affect efficacy and safety of the therapy. All information on the topic is likely to enhance the quality of pharmaceutical care.

## Impacts on practice

- It is important for optimal pharmaceutical care that the pharmacist has knowledge about interactions between food and citrus fruit juice and medicines to treat bladder dysfunction.
- Of the medicines to treat bladder dysfunction, only the effect of trospium was found to be significantly affected by food therefore the medication should be administered on an empty stomach.
- The majority of medicines to treat bladder dysfunction may potentially interact with grapefruit and other citrus fruit juice, and the effects of such interactions may be negative for patients.

## Introduction

Overactive bladder syndrome (OAB) is a condition where one or more of the symptoms such as pollakiuria, urgent need to urinate, nocturia and urinary incontinence [1] is observed. The syndrome may coexist with other diseases, such as diabetes, Parkinson’s disease, multiple sclerosis, stroke or spinal cord injuries, or be of idiopathic nature [2]. Its recorded incidence ranges between 7 and 27 % in males, and from 9 to 43 % in females [1]. According to the international population-based study performed in Europe and Canada, 12.8 % of females and 10.8 % of males suffer from OAB [3]. Especially urinary incontinence affects women more frequently. The frequency and severity of all symptoms tend to increase with age, and is two times more common in individuals over 65 years of age, compared to subjects below 45 [1, 3]. OAB is a condition that markedly deteriorates quality of life in patients [4]. Muscular and neural factors play a significant role in OAB pathophysiology [2]. The main group of drugs employed in OAB therapy includes the muscarinic receptor antagonists. The medicines inhibit the effect of acetylcholine on the muscular coat of the urinary bladder [5]. Adverse effects of anticholinergic drugs include xerostomia, constipation, visual disturbances, and rarely arrhythmia [4]. Medications such as mirabegron, a β_3_ receptor agonist, or botulinum toxin (in intradetrusor injections) have been introduced recently [6].

There are some recent developments around the treatment of bladder dysfunctions [7, 8]. New pharmacological targets can be found at the level of the urothelium, detrusor muscles, autonomic and afferent pathways, spinal cord and brain. Selected K^+^ ion channels potentially may provide therapeutic targets for bladder diseases. In the urinary bladder, activated K^+^ channels, in particular the large-conductance Ca^2+^-activated K^+^ channels (BK), prevents excessive excitability and contractility of urinary bladder smooth muscle. The BK channel seems to play a significant role in reducing both cholinergic- and purinergic-induced contractility and BK channel function alterations by specific drugs have been suggested to contribute to OAB occurrence. Activity in the serotonergic pathway can enlarge urine storage capability by facilitating the vesical sympathetic reflex pathway and inhibiting the parasympathetic voiding pathway. Thus, 5-HT receptor antagonists and reuptake inhibitors represent important targets for developing new OAB treatments. Alfa 1- and alfa 2-adrenoceptors seem to be also involved in micturition control. In addition, opioid receptors, and GABA-ergic systems open a wide range of possibilities. Recently, a relaxation of human detrusor smooth muscle induced by phosphodiesterase type 5 inhibitors with cGMP-, cAMP- and K^+^ channel-dependent signaling pathways involved have been reported. Nociceptin/orphanin FQ receptor agonists have been also suggested to be potentially effective new drugs for treating neurogenic urinary incontinence.

It is the pharmacist’s role to educate patients about proper medication administration schemes, and not to limit advice to drug–drug interactions, but to cover also drug–food and drug–particular food component interactions [9]. The appropriate mode of taking medicines is important for optimizing pharmacotherapy [10]. Knowledge about drug–food interactions is important for the safety and optimization of pharmacotherapy for bladder dysfunctions. A database on this topic will be helpful to pharmacists when counseling their patients, as a necessary element of pharmaceutical care.

## Aim of the review

In view of the increase in use of medicines for the dysfunctional bladder, it is important to investigate potential effects that food and fruit juice consumption may have on the pharmacokinetics and therapeutic effect of such medicines.

## Method

**Fig. 1:**
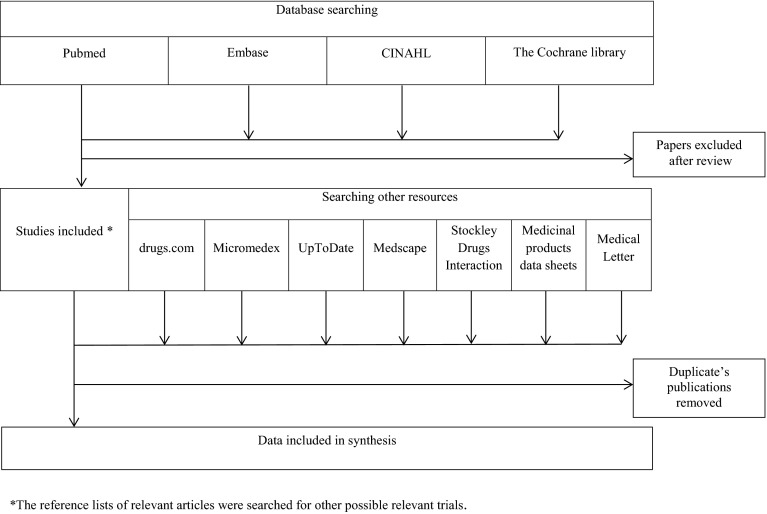
Flow chart of searching strategy

Four databases, PubMed, Embase, Cochrane and CINAHL covering reports from 1996 to 2015 have been searched with the following key words and phrases: drugs name for bladder dysfunctions treatments, urinary incontinence, food, fruit juices, grapefruit, pharmacokinetics and pharmacodynamics, plus drugs–food interaction. The search was limited to English papers and the reference lists quoted therein. Additionally, other resources such as drugs.com, Medscape, UpToDate, Micromedex, Medical Letter, as well as the electronic Stockley Drugs Interaction were also searched. Specific medicinal product datasheets were also included into analysis. Duplicated data were excluded. The search process is presented in Fig. 1. With all the materials collected, a critical review was carried out and the result is presented as a mini-review. Important information on drug–food interaction are presented in a tabular form (see Table 1). Practical recommendations for pharmacists and physicians how to solve food interaction problems for a particular drug are contained therein.

## Results and discussion

### Anticholinergic drugs

#### Darifenacin

Skerjanec et al. indicated that a single dose of darifenacin administered as extended-release tablets (30 mg) together with a high-fat meal not only did not affect the drug bioavailability, but resulted in a 22 % increase in C_max_ whereas T_max_ was prolonged by 3.3 h. Similarly, when administered in a multiple dose over 10 days, no food-related effect on its pharmacokinetic parameters was noted. Additionally, none of the adverse effects had increased, neither with respect to their number, nor intensity, for the drug administered with meals. Therefore, it can be either taken with food or on an empty stomach [11].

Darifenacin is metabolized in the liver by CYP 3A4 and 2D6 isoenzymes. Supposedly, for that reason a potential risk of drug–grapefruit juice interaction was reported, as the latter is a known CYP 3A4 inhibitor. Moreover, inhibited darifenacin metabolism might result in intensified adverse effects. However, the clinical importance of the interaction seems to be minor [12, 13].

#### Fesoterodine

Malhotra et al. [14] investigated the effect of food, namely high-fat, high-calorie breakfast, on the pharmacokinetics of 5-hydroxymethyl tolterodine (5-HMT), the main active metabolite of fesoterodine. The study included 16 healthy males, who were administered a single 8 mg dose of the drug. High-fat food resulted in insignificant increase in AUC, whereas C_max_ of the active metabolite increased by approximately 19 %. The remaining pharmacokinetic parameters, such as T_max_ or drug elimination parameters, did not change.

5-HMT is metabolized to carboxyl in a process involving CYP2D6 and CYP3A4 enzymes, carboxy-N-diisopropyl and N-diisopropyl metabolites, which do not have significant pharmacological activity. The respective mean values for C_max_ and AUC were respectively found to be 1.7 and twice higher in individuals showing a weak metabolizing activity of CYP2D6, as compared to subjects with a high metabolic potency of the isoenzyme [15]. For this reason, Malhorta et al. [16] studied the pharmacokinetic parameters of a single administration of fesoterodine given as extended-release tablets at doses of 4, 8 and 12 mg to 24 healthy volunteers, 16 of whom showed high metabolic potency, and 8 low metabolic potency of CYP2D6. Additionally, for the dose of 8 mg, the effect of high-calorie and high-fat food on the pharmacokinetics of 5-HMT drug was studied; AUC and C_max_ were found to increase by 12 and 29 %, respectively, due to consumed food, though such changes were not considered to be of clinical significance. Similarly, none meal-related effect was demonstrated for CYP2D6 activity. 5-HMT C_max_ and AUC were approximately two times higher in individuals with low CYP2D6 metabolic potency, yet the activity of the isoenzyme was found to affect neither T_max_, renal clearance, nor half-life of the medication. Only for doses above 8 mg, an increase in the prevalence of xerostomia was observed, though the changes were mild and well-tolerated by the study participants. Based on the above results, the drug can be taken irrespectively of meals.

The use of moderate CYP3A4 inhibitors, i.e. grapefruit juice and other grapefruit-based products, may result in increased serum concentration levels of the active metabolite of fesoterodine 5-HMT, which is partially metabolized by the isoenzyme. Since 5-HMT is also metabolized by CYP2D6, the clinical importance of the interaction may be higher in patients with a low metabolic potency for CYP2D6 isoenzyme, i.e. approximately for 7 % of Caucasians, and below 2 % of Asians and people of African descent, in whom the CYP3A4-dependent metabolic pathway is more significant for the further drug processing in the body. Thus, increased fesoterodine activity should be taken into account whenever the drug administration coincides with grapefruit elements in the patients diet. Such adverse effects of the drug may manifest itself by irregular heartbeat, blurred vision, difficult urination, xerostomia, headache, somnolence, dizziness, gastrointestinal problems or constipation [12, 15].

#### Oxybutynin

Sathyan et al. [17] investigated the effect of food on bioavailability of oxybutynin (15 mg) administered as a single extended-release dose to 50 healthy volunteers. Their meal consisted of high-fat breakfast, and they studied the effect on the drug pharmacokinetics and its active metabolite. No significant food-related effect on the above parameters was demonstrated.

Lukkari et al. [18] performed a similar study on the effect of high-fat food on the pharmacokinetic properties of oxybutynin and N-deethyloxybutynin, its active metabolite. The medication was administered as a single 10 mg dose in extended-release tablets to a group of 23 healthy volunteers, including 12 women and 11 men. High-fat breakfast was found not to change oxybutynin AUC, though it triggered a significant increase in AUC, reportedly by approximately 20 %, for its active metabolite. The food doubled the increase in C_max_ for oxybutynin and N-deethyloxybutynin alike. Significantly prolonged T_max_ for the metabolite of the drug was attributed to meal. Though no significant differences in the prevalence of adverse effects was observed, for the medication taken after a meal, a decrease in saliva secretion was found to be higher than when the drug was administered on an empty stomach.

Lukkari et al. [19] investigated the effect of food on the prevalence of drug related adverse effects. The authors confirmed that a meal resulted in a significant increase in C_max_ both for the drug and its metabolite, and increased AUC for N-deethyloxybutynin. However, for the medication administered 2 h after a meal, the amount of produced saliva was observed to significantly decrease, as compared to oxybutynin administration on an empty stomach.

For oxybutynin given as a solution, a meal resulted in prolonged T_max_ and 25 % increase in AUC [20].

Observations as above, give ground to surmise that as food-related changes in the drug pharmacokinetics were of no clinical significance, the medication may be administered irrespectively of meals. Nevertheless, administration of oxybutynin, especially in its extended-release form, one hour before a meal allows for achieving the drug concentration value that shows only slight variations between the employed doses; this may be of importance in improved tolerance of the medications in patients who suffer from limited saliva secretion during the therapy.

As the oxybutynin is metabolized by CYP 3A4, caution is recommended when simultaneously consuming grapefruit juice, a known inhibitor of the enzyme. Simultaneous consumption of oxybutynin and grapefruit juice may potentially increase the risk of adverse effects associated with the medication. The clinical significance of the interaction remains unknown [21].

#### Solifenacin

Uchida et al. [22] studied the effect of food on the pharmacokinetics of solifenacin (10 mg). Twenty-three healthy men were divided into two groups. The first group received the drug on an empty stomach together with 180 ml of water, while the second group took the medication 5 min following a standard high-fat meal of 1000 kcal, with approximately 50–60 % of the total calories originating from fats, 15 % from proteins and approximately 25 % from carbohydrates. No changes were noted in the pharmacokinetic parameters of the medication administered with the meal.

Solifenacin is metabolized in the liver, in the process where CYP3A4 isoenzyme is predominantly involved. Thus, grapefruit juice which inhibits the isoenzyme activity causes blood-solifenacin concentration to increase, and may increase the risk of toxicity [23].

#### Tolterodine

Ollson et al. [24] investigated the effect of medium-fat breakfast on the pharmacokinetic parameters of tolterodine and its active metabolite 5-hydroxymethyl tolterodine (5-HMT). To meet the study objective 23 healthy volunteers with normal CYP2D6 isoenzyme activity were administered a single dose of tolterodine, namely 2 mg of immediate-release form (IR). For tolterodine, the meal resulted in increased AUC and C_max_ by 53 and 49 %, respectively, while for the active metabolite of 5-HMT, food was found not to change the drug pharmacokinetic parameters. In spite of significant alterations in bioavailability of tolterodine, its clinical effect was not affected.

In another study by Ollson et al. [25] the effect of food on the pharmacodynamics of tolterodine administered 2 × 40 mg as extended-release capsules (ER) and its metabolite 5-HMT was studied. Seventeen healthy volunteers, 3 women and 14 men, of whom one male demonstrated a decreased activity of CYP2D6 isoenzyme, received the medicine either on an empty stomach, or after a high-fat meal. No effect of food on the bioavailability of the ER form of the drug was observed.

#### Trospium

Food, especially high-fat products, cause C_max_ and AUC of trospium to decrease by approximately 15–20 % [26].

Doroshyenko et al. [27] reported twenty-four healthy males who have received two dragees of trospium at the dose of 20 mg either on an empty stomach, or after a high-fat meal. In consequence of administering the drug with a meal, a significant decrease in C_max_ occurred, specifically from 9.2 ng/ml on an empty stomach to 1.3 ng/ml after a meal on average. AUC of the drug was reduced by over 70 %, from 87.2 ng × h/ml on an empty stomach to 20.1 ng × h/ml after a meal. The food resulted in decreasing T_max_ from 5 h on an empty stomach to 3.3 h after a meal [27].

Administration of trospium as extended-release capsules together with a high-fat meal caused a drop in AUC and C_max_ by o 35 and 60 %, respectively [28].

Taking trospium with a high-fat meal resulted in a decrease in the drug absorption, with a decrease of the AUC and C_max_ parameters by 70–80 %, as compared to its administration on an empty stomach [26]. This is also confirmed by the drugs.com database [29].

### β_3_ adrenoceptor agonist

#### Mirabegron

Lee et al. [30] investigated the effect of high-fat and low-fat breakfast on the pharmacokinetic parameters of mirabegron taken as OCAS tablets (orally controlled absorption system) administered at doses of 50 and 100 mg to 38 healthy men, either 30 min after a meal, or on an empty stomach.

For a high-fat meal and the drug given at the dose of 50 mg, a 45 % decrease was noted in C_max_ and a 17 % drop in AUC. The use of mirabegron at the dose of 100 mg with the same meal resulted in decreasing C_max_ and AUC by 39 and 18 %, respectively.

A low-fat meal also resulted in a decrease in C_max_ and AUC; at a 50 mg dose by 75 % and 51 %, respectively, and at the dose of 100 mg by 64 and 47 %.

Changes in T_max_ were independent of the amount of fat in the meal; in general, food resulted in prolonged T_max_ that by 0.9 h and 1.5–2 h for the dose of 50 and 100 mg, respectively.

The use of mirabegron as OCAS tablets together with a meal resulted in significant changes in the pharmacokinetic parameters that were independent of the dose, but dependent on the meal fat content. A low-fat meal triggered a significantly higher reduction in bioavailability of the drug as compared to a high-fat meal. Such significant differences in the drug pharmacokinetics contributed to the meal effect are not, however, important with respect to the drug effectiveness, and therefore mirabegron may be administered irrespectively of meals [31].

Overall it is clear that there are gaps in the knowledge on interaction with medicines for bladder dysfunction, especially with respect to the consequences of food–drug interactions. Data to guide clinical recommendations is scarce. Heuberger [32] and Paśko et al. [33] described several reason for that, such as measurement difficulties, hardly available proper samples, as well as the lack of study framework and little research interest. Few reserachers acknowledge the importance of the problem, its clinical significance, cost, and overall impact on the population.

**Table 1:** Interactions of drugs employed in OAB with food and recommendations on their administration

Drug	Drug–food interaction	Recommendations
Darifenacin	Potential interaction with grapefruit juice	May be taken irrespectively of a meal Exercise caution while consuming grapefruit juice during therapy
Fesoterodine	Potential interaction with grapefruit juice	May be taken irrespectively of a meal Avoid consumption of grapefruit juice during therapy
Oxybutynin	Potential interaction with grapefruit juice	May be taken irrespectively of a meal In patients manifesting disturbances of saliva secretion during therapy, taking the drug 0.5–1 h prior to a planned meal is recommended Exercise caution while consuming grapefruit juice during therapy
Solifenacin	Potential interaction with grapefruit juice	May be taken irrespectively of a meal Grapefruit juice should not be consumed during solifenacin therapy
Tolterodine	Data unavailable	May be taken irrespectively of a meal
Trospium	Food, especially high in fats, decreases absorption of the drug	To be taken 1 h prior to a meal or on an empty stomach
Mirabegron	Data unavailable	May be taken irrespectively of a meal

## Conclusion

An appropriate mode of administering medications employed in overactive bladder syndrome and other lower urinary tract dysfunctions may result in limiting the occurrence of adverse effects and optimal use of the lowest possible doses of the drugs. As far as food is concerned most drugs can be taken irrespectively of meals. Only trospium is to be taken either on empty stomach, or an hour before the meal. As most of the described medications, such as darifenacin, fesoterodine, oxybutynin, and solifenacin, are metabolized in the liver by CYP 3A, their potential to interact with grapefruit juice cannot be neglected. As reported by Mazi-Kotwal et al. [12] and Baily et al. [34] the clinical effect of such interaction was determined as ‘moderate’ or ‘intermediate’. Specific medicinal substances, or supplements, consumed with food may significantly affect the efficacy and safety of the therapy. Gaps in knowledge on interactions especially with respect to the consequences of food–drug interactions are evident.
